# Possible benefits of singing to the mental and physical condition of the elderly

**DOI:** 10.1186/1751-0759-8-11

**Published:** 2014-05-21

**Authors:** Katsuhisa Sakano, Koufuchi Ryo, Yoh Tamaki, Ryoko Nakayama, Ayaka Hasaka, Ayako Takahashi, Shukuko Ebihara, Keisuke Tozuka, Ichiro Saito

**Affiliations:** 1 Department of Pathology, Tsurumi University School of Dental Medicine, 2-1-3 Tsurumi-Ku, Yokohama 230-8501, Japan; 2 PREMEDiCO Co, Ltd. 4 F Chushin Build. 3-3-5, Chiyoda-ku, Tokyo 101-0047, Uchikanda, Japan; 3 Department of Health and Welfare Services, National Institute of Public Health, 2-3-6 Minami, Wako-shi, Saitama 351-0197, Japan; 4 Chiyoda Paramedical Care Clinc, 2F Chushin Build. 3-3-5, Chiyoda-ku, Tokyo 101-0047, Uchikanda, Japan; 5 Daiichikosho Co, Ltd. 5-5-26 Kitashinagawa, Shinagawa-ku, Tokyo 141-8701, Japan

## Abstract

**Background:**

The evaluation and management of stress are important for the prevention of both depression and cardiovascular disease. In addition, the maintenance of the oral condition of the elderly is essential to enable them to stay healthy, especially to prevent aspiration pneumonia and improve mental health in an aging society. Therefore, we examined the efficacy of singing on the oral condition, mental health status, and immunity of the elderly to determine if singing could contribute to the improvement of their physical condition.

**Methods:**

Forty-four subjects (10 men, 34 women), aged 60 years or older, participated in this study. The efficacy of singing on mental health status and immunocompetence was examined by swallowing function, oral condition, blood, and saliva tests, as well as through questionnaires taken before and after singing.

**Results:**

The results showed that the amount of saliva increased and the level of cortisol, a salivary stress marker, decreased after singing. The Visual Analog Scale (VAS) scores for feeling refreshed, comfortable, pleasurable, light-hearted, relieved, and relaxed; the tension and confusion subscale score; and the total mood disturbance (TMD) score of the Profile of Mood States (POMS) all showed improvements. Furthermore, the same tendencies were shown regardless of whether or not the subjects liked singing.

**Conclusions:**

Our results suggest that singing can be effective in improving the mental health and oral condition of the elderly.

## Background

Many recent studies have reported that stress-associated factors are detrimental to overall health [1,2]. According to a survey conducted by the Japanese Cabinet Office, approximately 60% of people experience stress in their daily lives for various reasons, including work, school, and relationships. Furthermore, the number of stress-related depression and suicide cases is increasing every year [3,4]. An epidemiological study showed that people who suffered from daily stress had higher incidences of cerebrovascular disease and ischemic heart disease; therefore, the evaluation and management of stress is important for the prevention of depression and cardiovascular disease [5].

The immune system is known to be affected by stress: both the hypothalamic-pituitary-adrenal (HPA) system and the locus coeruleus-noradrenalin system, which carry out endocrine reactions, are activated by stress. In the HPA system, corticotropin-releasing hormone released from the hypothalamus acts on the pituitary gland, which induces the secretion of the adrenocorticotrophic hormone from the pituitary gland. The adrenocorticotrophic hormone then stimulates the release of cortisol from the adrenal cortex. In the locus coeruleus-noradrenalin system, which originates from the locus coeruleus, the autonomic nervous system, especially the sympathetic nervous system, is activated by noradrenalin. This activation leads to both the secretion of noradrenalin from nerve endings, which then affects target tissues, and the release of catecholamine from the adrenal medulla. The levels of stress markers, such as lysozyme, cortisol, chromogranin A, amylase, and secretory immunoglobulin A, can now be measured in saliva.

In addition, the Japanese National Institute of Population and Social Security Research projected that the population will age rapidly in the coming 10 years and that, around the year 2040, approximately 30% of the entire population will be aged 65 years or older [6]. Recent studies have shown that in such an aging society, maintaining good oral condition is essential for enabling the elderly to stay healthy, especially for the prevention of aspiration pneumonia and the improvement of mental health [7-9].

There are few reports of objective evaluations of the effects of singing, such as the effects on physical conditions and mental health status [10-12]. This lack of information prompted us to conduct a study to evaluate the effects of singing on physical conditions, including oral condition.

### Study design

This prospective, open-label study was conducted according to the ethical principles of the Declaration of Helsinki. All subjects read and signed a written informed consent form, and the study was approved in advance by the Institutional Review Board of the Chiyoda Paramedical Care Clinic.

### Participants

Forty-four subjects (10 men, 34 women), recruited by flyers and the Internet, aged 60 years or older, participated in this study in the Chiyoda Paramedical Care Clinic. The exclusion criteria were as follows: continuous use of medication for respiratory or cardiovascular diseases; regular use of supplements for mental health or immune status that might affect the outcome of this study; medical history of serious disease of the heart, liver, kidney, lung, or digestive organs (including gastrectomy) or in the blood, endocrine, or metabolic systems; current medical treatment; difficulty in keeping appointments; depression, a mentally unbalanced state, or frequent psychogenic reactions; or ineligibility due to other factors at the discretion of the investigator.

### Procedure

This was an open-label study. The subjects were requested to not eat or drink in excess the night before the test but rather to eat and drink as they normally would, as poor physical condition or fullness can affect the amount of saliva secreted. The consumption of alcohol, the consumption of substances likely to affect salivary stress markers (green tea, coffee, black tea, cola, and energy drinks that contain a high level of caffeine), excessive exercise (physical stress can affect salivary stress markers), and the ingestion of antihistamines (known to inhibit salivary secretion) were prohibited from 9 p.m. the previous night until completion of the test. Additionally, the consumption of any substance other than water was prohibited, as other substances can affect salivary stress markers. Subjects were required to arrive 40 minutes before the test and were seated quietly in the testing room 30 minutes prior to the test, during which time they were asked to complete the pre-test VAS and POMS questionnaires. Subjects were measured for height, weight, blood pressure, and heart rate. Unstimulated saliva collection, a Saxon test, stimulated saliva collection, and blood collection were performed in that order. The subjects then sang three songs consecutively. They selected songs that they could sing in entirety. The mean singing duration was 3 minutes 50 seconds, and there was no notable deviation in the singing time (3SD). After singing, saliva and blood samples were collected in the same manner as that used for the pre-test, and the subjects once again completed the POMS and VAS questionnaires. We prepared a questionnaire sheet with the questions “Do you like singing?” and “Did you sing well?” and responses of “Yes” or “No.”

### Measurements

Height, weight, blood pressure, and pulse rate measurement and saliva and blood collection were all performed at the Chiyoda Paramedical Care Clinic.

The VAS scores for feeling refreshed, comfortable, pleasurable, light-hearted, relieved, and relaxed were rated on a 100-mm vertical line anchored at the bottom (zero) with the statement “I feel nothing” and at the top (100 mm) with the statement “I feel great.” Participants were asked to look at the line and think about how their statement was affected by their singing. There were small horizontal lines along the vertical line at each centimeter interval, and participants had to mark where they felt they were along the spectrum.

In the Saxon test, saliva production is measured by weighing a folded, sterile, gauze pad both before and 2 min after chewing without swallowing; the low-normal value is 2 g [13].

In the Repetitive Saliva Swallowing Test (RSST), the patient was required to swallow saliva as often as possible while in a sitting position for 30 sec, during which time the number of clear palpations of the laryngeal prominence and elevations of the hyoid bone were counted.

The levels of the salivary markers lysozyme, chromogranin A, cortisol, secretory immunoglobulin A, and amylase were measured. Blood samples were measured for dehydroepiandrosterone sulfate (DHEA-s) levels and natural killer (NK) cell activity. To measure swallowing and oral condition, the RSST, bite force test, oral moisture content test, unstimulated saliva test, stimulated saliva test, and Saxon test were performed. The saliva and blood collections were performed in the clinic between 2:00 and 4:00 p.m. to minimize diurnal variation.

### Statistical analysis

Results were considered statistically significant at the level of p < 0.05, and all tests were two-tailed. Data in the text are presented as the mean ± S.D. A two-way repeated measures analysis of variance (ANOVA) was performed to test for the main effects corresponding to Preference (“like singing” or “dislike singing”) and Time (“before singing” and “after singing”), as well as the interaction between the two. These analyses were performed using IBM SPSS (Statistical Package for the Social Sciences) Statistics Version 19 software (IBM Japan Inc., Tokyo, Japan).

## Results

Forty-four subjects (10 men and 34 women), with a mean age of 64.1 ± 3.9 years, participated in this study. All subjects met the inclusion criteria and provided informed consent. All 44 subjects completed the study, and the characteristics of the subjects are shown in Table 1. Based on the combination of answers in the pre- and post-questionnaires, seventy-three percent of the subjects (32 subjects) provided the response combination of “I like singing” and “I sang well” (hereafter, this combination is referred to as “like singing”), and twenty-seven percent (12 subjects) provided the response combination of “I dislike singing” and “I did not sing well” (hereafter, this combination is referred to as “dislike singing”). No significant differences were found with respect to gender, age, height, and weight between the “like singing” and “dislike singing” groups.

**Table 1:** Subject characteristics

**Items**		**Like singing**	**Diskike singing**	**p-value**
Number of subjects		32	12	–
Sex	Male	7	3	0.826
	Female	25	9	
Age		64.3 ± 4.4	63.4 ± 2.4	0.727
Heights (cm)		157.5 ± 8.1	157.6 ± 7.7	0.580
Weight (kg)		55.94 ± 11.1	62.7 ± 11.3	0.067

Chi-square test was used to compare the sex and Mann-Whitney U test was used to compare the age, height, and weight of the subjects who like singing and those who dislike singing.

### Blood pressure and pulse rates

The blood pressure and pulse rate results are shown in Tables 2 and 3. The two-way repeated measures ANOVA analysis indicated that systolic blood pressure was significantly increased (p < 0.01, main effect) after singing (130.0 ± 23.8 bpm) compared with before singing (126.0 ± 23.8 mmHg). The two-way repeated measures ANOVA also revealed that pulse rates were significantly decreased (p < 0.01, main effect) after singing (74.6 ± 8.5 bpm) compared with before singing (77.3 ± 9.8 bpm).

**Table 2:** Physiological parameters

**Items**	**Group**	**Before singing**	**After singing**
Systolic blood pressure (mmHg)	Total	126.0 ± 23.8	130.0 ± 23.8
	Like singing	129.4 ± 25.2	132.1 ± 24.2
	Diskike singing	116.8 ± 17.3	124.2 ± 22.9
Diastolic blood pressure (mmHg)	Total	73.4 ± 15.2	75.3 ± 15.2
	Like singing	74.7 ± 15.2	76.3 ± 14.8
	Diskike singing	69.9 ± 15.3	72.8 ± 16.6
Pulse rate (bpm)	Total	77.3 ± 9.8	74.6 ± 8.5
	Like singing	75.2 ± 9.4	72.9 ± 7.6
	Diskike singing	83.0 ± 8.7	79.0 ± 9.5

Values represent the mean ± SD.

**Table 3:** Results of the two—way repeated measures ANOVA for the physiological parameters

**Items**	**Results of two–way repeated measures ANOVA**					
	**Variables**	**SS (Type III)**	**DF**	**MF**	**F**	**p-value**
Systolic blood pressure (mmHg)	Time	3778.705	1	3778.705	15.089	<0.001
	Time* like or dislike	0.250	1	0.250	0.001	0.975
	Like or dislike	2825.875	1	2825.875	4.338	0.043
Diastolic blood pressure (mmHg)	Time	87.547	1	87.547	1.811	0.186
	Time* like or dislike	8.002	1	8.002	0.166	0.686
	Like or dislike	292.517	1	292.517	0.702	0.407
Pulse rate (bpm)	Time	172.163	1	172.163	11.987	0.001
	Time* like or dislike	12.891	1	12.891	0.898	0.349
	Like or dislike	843.857	1	843.857	6.159	0.017

*interaction.

The results of the two-way repeated measures ANOVA showed a significant main effect of preference (like or dislike of singing) on both the systolic blood pressure and pulse rate. The systolic blood pressure of the “dislike singing” group was lower than that in the “like singing” group before singing; and the pulse rate of the “dislike singing” group was higher than that in the “like singing” group before singing.

### Swallowing and oral condition

The swallowing and oral condition results are shown in Tables 4 and 5. The unstimulated saliva test results showed a significant increase in saliva production (p < 0.01, main effect) after singing (4.0 ± 2.2 ml) compared with before singing (3.3 ± 2.0 ml).

**Table 4:** Swallowing and oral function

**Items**	**Group**	**Before singing**	**After singing**
RSST (time)	Total	4.1 ± 1.8	4.3 ± 1.8
	Like singing	4.2 ± 1.7	4.6 ± 1.6
	Diskike singing	3.9 ± 2.1	3.5 ± 2.1
Bite force test (KN)	Total	0.166 ± 0.108	0.171 ± 0.098
	Like singing	0.184 ± 0.123	0.192 ± 0.106
	Diskike singing	0.138 ± 0.079	0.139 ± 0.079
Oral moisture content test	Total	28.3 ± 1.8	28.8 ± 1.8
	Like singing	28.3 ± 1.8	28.9 ± 1.9
	Diskike singing	28.2 ± 1.9	28.4 ± 1.6
Unstimulated saliva test (ml)	Total	3.3 ± 2.0	4.0 ± 2.2
	Like singing	3.3 ± 1.9	3.9 ± 2.3
	Diskike singing	3.3 ± 2.2	4.3 ± 2.1
Saxon test (g)	Total	4.2 ± 1.8	4.3 ± 1.9
	Like singing	4.3 ± 1.8	4.4 ± 2.0
	Diskike singing	3.9 ± 1.8	4.3 ± 1.8
Stimulated saliva test (ml)	Total	14.9 ± 5.3	15.1 ± 5.6
	Like singing	14.9 ± 5.8	14.9 ± 6.1
	Diskike singing	15.0 ± 3.7	15.5 ± 4.1

Values represent the ± SD.

23 people for the bite force test.

33 people for the oral moisture content test.

**Table 5:** Results of the two–way repeated measures ANOVA for swallowing and oral function

**Items**	**Results of two-way repeated measures ANOVA**					
	**Variables**	**SS (Type III)**	**DF**	**MF**	**F**	**p–value**
RSST (time)	Time	0.000	1	0.000	0.001	0.978
	Time* like or dislike	2.955	1	2.955	4.716	0.036
	Like or dislike	7.398	1	7.398	1.307	0.259
Bite force test (KN)	Time	0.000	1	0.000	0.135	0.717
	Time* like or dislike	0.000	1	0.000	0.065	0.801
	Like or dislike	0.027	1	0.027	1.385	0.252
Oral moisture content test	Time	2.291	1	2.291	0.743	0.395
	Time* like or dislike	0.627	1	0.627	0.203	0.655
	Like or dislike	1.346	1	1.346	0.374	0.545
Unstimulated saliva test (ml)	Time	10.611	1	10.611	15.872	<0.001
	Time* like or dislike	0.506	1	0.506	0.757	0.389
	Like or dislike	1.086	1	1.086	0.131	0.719
Saxon test (g)	Time	0.796	1	0.796	1.189	0.282
	Time* like or dislike	0.556	1	0.556	0.831	0.367
	Like or dislike	1.287	1	1.287	0.203	0.655
Stimulated saliva test (ml)	Time	1.266	1	1.266	0.394	0.533
	Time* like or dislike	1.068	1	10.68	0.333	0.567
	Like or dislike	2.461	1	2.461	0.043	0.837

*interaction.

With regard to the unstimulated saliva test results, there was no significant interaction between preference and time.

The results of the two-way repeated measures ANOVA revealed an interaction between the two main factors (time and like or dislike of singing) in the RSST.

The results of the simple main effects in the RSST are shown in Table 6. The RSST results showed a tendency to increase (p < 0.061) after singing (4.6 ± 1.6 times) compared with before singing (4.2 ± 1.7 times) in the “like singing” group.

**Table 6:** RSST

**Items**	**Group**	**Number of subjects**	**Before singing**	**After singing**	p–value*
RSST (time)	Total	44	4.1 ± 1.8	4.3 ± 1.8	0.323
	Like singing	32	4.2 ± 1.7	4.6 ± 1.6	0.061
	Diskike singing	12	3.9 ± 2.1	3.5 ± 2.1	0.132

Values represent the mean ± SD.

*Wilcoxon signed rank test.

### Blood measurement

The results of the blood measurements are shown in Tables 7 and 8. No significant difference was found in any of the measurements.

**Table 7:** Blood measurement

**Items**	**Group**	**Before singing**	**After singing**
DHEA–S (*μ* g/dL)	Total	106.1 ± 79.3	106.3 ± 77.9
	Like singing	111.3 ± 79.4	111.0 ± 77.3
	Diskike singing	92.2 ± 80.9	93.5 ± 81.7
NK cell activity (%)	Total	32.9 ± 14.4	32.8 ± 14.6
	Like singing	31.3 ± 13.8	31.5 ± 14.6
	Diskike singing	37.3 ± 15.5	36.3 ± 14.5

Values represent the mean ± SD.

**Table 8:** Results of the two–way repeated ANOVA for blood measurement

**Items**	**Results of two-way repeated measures ANOVA**					
	**Variables**	**SS (Type III)**	**DF**	**MF**	**F**	**p–value**
DHEA–S (*μ* g/dL)	Time	4.830	1	4.830	0.074	0.978
	Time* like or dislike	11.375	1	11.375	0.174	0.036
	Like or dislike	5870.000	1	5870.000	0.471	0.496
NK cell activity (%)	Time	2.125	1	2.125	0.126	0.724
	Time* like or dislike	5.625	1	5.625	0.334	0.566
	Like or dislike	503.296	1	503.296	1.260	0.268

*interaction.

### Stress markers in saliva

The changes in stress markers are shown in Tables 9 and 10. A significant decrease (p < 0.01, main effect) in cortisol was noted after singing (0.09 ± 0.05 ng/ml) compared with the amount present before singing (0.12 ± 0.07 ng/ml). The amount of secretory immunoglobulin A (S-IgA) was significantly decreased (p < 0.01, main effect) before singing (28.8 ± 16.3 ng/ml) compared with the amount after singing (17.7 ± 9.3 ng/ml).

**Table 9:** Stress markers in saliva

**Items**	**Group**	**Before singing**	**After singing**
Lysozyme (*μ* g/mL)	Total	13.6 ± 11.7	13.8 ± 9.6
	Like singing	13.7 ± 12.8	13.5 ± 9.3
	Diskike singing	13.3 ± 8.5	14.6 ± 10.7
Chromogranin A (ng/mL)	Total	10.8 ± 16.6	11.6 ± 16.2
	Like singing	10.2 ± 12.8	11.0 ± 14.5
	Diskike singing	12.6 ± 24.7	13.4 ± 20.7
Cortisol (ng/mL)	Total	0.12 ± 0.07	0.09 ± 0.05
	Like singing	0.12 ± 0.07	0.09 ± 0.05
	Diskike singing	0.12 ± 0.06	0.10 ± 0.05
S–IgA (ng/mL)	Total	28.8 ± 16.3	17.7 ± 9.3
	Like singing	28.1 ± 17.1	17.5 ± 9.2
	Diskike singing	30.6 ± 14.3	18.5 ± 10.0
Amylase (ng/mL)	Total	417.8 ± 153.4	430.7 ± 170.3
	Like singing	414.4 ± 163.1	439.1 ± 186.7
	Diskike singing	426.8 ± 130.0	408.2 ± 119.8

Values represent the mean ± SD.

**Table 10:** Results of the two-way repeated measures ANOVA on stress markers in saliva

**Items**	**Results of two-way repeated measures ANOVA**					
	**Variables**	**SS (Type III)**	**DF**	**MF**	**F**	**p–value**
Lysozyme (*μ* g/mL)	Time	4.754	1	4.754	0.185	0.670
	Time* like or dislike	9.900	1	9.900	0.385	0.539
	Like or dislike	2.075	1	2.075	0.010	0.921
Chromogranin A (ng/mL)	Time	10.533	1	10.533	0.169	0.683
	Time* like or dislike	0.019	1	0.019	0.000	0.986
	Like or dislike	100.137	1	100.137	0.207	0.652
Cortisol (ng/mL)	Time	0.013	1	0.013	10.124	0.003
	Time* like or dislike	0.000	1	0.000	0.069	0.795
	Like or dislike	0.000	1	0.000	0.001	0.974
S–IgA (ng/mL)	Time	2245.723	1	2245.723	35.614	<0.001
	Time* like or dislike	8.977	1	8.977	0.142	0.708
	Like or dislike	54.564	1	54.564	0.185	0.670
Amylase (ng/mL)	Time	165.859	1	165.859	0.026	0.872
	Time* like or dislike	8216.001	1	8216.001	1.308	0.259
	Like or dislike	1480.858	1	1480.858	0.031	0.860

*interaction.

No significant difference in the results of any saliva test was found between the two groups of subjects who responded that they “like singing” and “dislike singing.” With regard to the stress markers in saliva, there was no significant interaction between preference and time.

### VAS

The VAS results for feeling refreshed, comfortable, pleasurable, light-hearted, relieved, and relaxed are shown in Tables 11 and 12.

**Table 11:** Results of VAS

**Items**	**Group**	**Before singing**	**After singing**
Refreshed	Total	47.5 ± 21.7	65.4 ± 19.5
	Like singing	49.7 ± 21.2	69.9 ± 15.8
	Diskike singing	41.5 ± 22.9	53.3 ± 23.7
Comfortable	Total	45.7 ± 22.9	68.7 ± 16.1
	Like singing	48.1 ± 23.2	71.0 ± 15.7
	Diskike singing	39.3 ± 21.8	62.6 ± 16.3
Pleasurable	Total	48.7 ± 23.4	63.8 ± 20.4
	Like singing	51.3 ± 23.0	68.3 ± 18.9
	Diskike singing	41.6 ± 24.1	51.6 ± 20.0
Light hearted	Total	46.1 ± 22.2	71.6 ± 16.9
	Like singing	47.0 ± 21.6	76.1 ± 12.7
	Diskike singing	43.8 ± 24.6	59.5 ± 20.9
Relieved	Total	41.1 ± 19.2	78.1 ± 13.4
	Like singing	41.6 ± 19.5	80.2 ± 11.6
	Diskike singing	39.8 ± 19.2	72.5 ± 16.8
Relaxed	Total	41.6 ± 19.7	75.4 ± 16.3
	Like singing	44.3 ± 20.8	79.1 ± 13.5
	Diskike singing	34.7 ± 15.1	65.5 ± 19.3

Values represent the mean ± SD.

**Table 12:** Results of the two–way repeated measures ANOVA for VAS

**Items**	**Results of two-way repeated measures ANOVA**					
	**Variables**	**SS (Type III)**	**DF**	**MF**	**F**	**p–value**
Refreshed	Time	4468.655	1	4468.655	15.785	<0.001
	Time* like or dislike	300.895	1	300.895	1.066	0.308
	Like or dislike	2679.980	1	2679.980	5.164	0.028
Comfortable	Time	9308.041	1	9308.041	39.507	<0.001
	Time* like or dislike	0.691	1	0.691	0.003	0.957
	Like or dislike	1291.877	1	1291.877	2.409	0.128
Pleasurable	Time	3197.558	1	3197.558	12.418	0.001
	Time* like or dislike	209.954	1	209.954	0.815	0.372
	Like or dislike	3058.825	1	3058.825	4.697	0.036
Light hearted	Time	8766.548	1	8766.548	33.403	<0.001
	Time* like or dislike	791.963	1	791.963	3.018	0.090
	Like or dislike	1711.260	1	1711.260	3.615	0.064
Relieved	Time	22189.857	1	22189.857	98.885	<0.001
	Time* like or dislike	153.296	1	153.296	0.683	0.413
	Like or dislike	398.322	1	398.322	1.225	0.275
Relaxed	Time	18846.943	1	18846.943	85.365	<0.001
	Time* like or dislike	71.097	1	71.097	0.322	0.573
	Like or dislike	2349.738	1	2349.738	6.028	0.018

*interaction.

The results of the two- way repeated measures ANOVA indicated a significant main effect of time (before and after singing) on “refreshed,” “comfortable,” “pleasurable,” “light-hearted,” “relieved,“ and “relaxed” after singing compared with before singing.

The results of the two-way repeated measures ANOVA showed a significant main effect of preference (like or dislike of singing) on “refreshed,” “pleasurable,” and “relaxed.” The results for “refreshed,” “pleasurable,” and “relaxed” feelings in the “dislike singing” group were lower than those in the “like singing” group before singing.

### POMS

The POMS data are shown in Tables 13 and 14. A significant main effect of time (after singing vs. before singing) was noted for “tension,” “confusion,” and “total mood disturbance (TMD),” and there was no significant interaction between preference and time in any of the subscales or in the TMD of the POMS.

**Table 13:** Results of POMS

**Items**	**Group**	**Before singing**	**After singing**
Tension	Total	5.2 ± 3.8	2.3 ± 2.3
	Like singing	4.3 ± 2.9	2.0 ± 2.3
	Diskike singing	7.7 ± 4.9	2.9 ± 2.2
Depression	Total	1.2 ± 1.7	0.6 ± 1.3
	Like singing	1.2 ± 1.9	0.7 ± 1.3
	Diskike singing	1.0 ± 1.2	0.5 ± 1.2
Anger–Hostility	Total	0.6 ± 1.2	0.2 ± 0.7
	Like singing	0.5 ± 1.2	0.2 ± 0.5
	Diskike singing	0.9 ± 1.2	0.3 ± 1.2
Vigor	Total	6.9 ± 4.0	7.5 ± 5.3
	Like singing	7.4 ± 4.1	8.3 ± 5.7
	Diskike singing	5.5 ± 3.4	5.5 ± 3.5
Fatigue	Total	2.1 ± 3.1	1.6 ± 2.3
	Like singing	2.1 ± 3.2	1.5 ± 2.4
	Diskike singing	2.3 ± 2.9	1.8 ± 2.1
Confusion	Total	4.4 ± 1.6	3.4 ± 1.6
	Like singing	4.3 ± 1.7	3.4 ± 1.7
	Diskike singing	4.8 ± 1.3	3.3 ± 1.3
TMD	Total	6.6 ± 9.8	0.6 ± 9.4
	Like singing	4.9 ± 9.7	-0.5 ± 10.2
	Diskike singing	11.1 ± 8.8	3.3 ± 6.2

Values represent the mean ± SD.

**Table 14:** Results of the two–way repeated measures ANOVA for POMS

**Items**	**Results of two-way repeated measures ANOVA**					
	**Variables**	**SS (Type III)**	**DF**	**MF**	**F**	**p–value**
Tension	Time	213.818	1	213.818	31.386	<0.001
	Time* like or dislike	27.273	1	27.273	4.003	0.052
	Like or dislike	81.939	1	81.939	7.490	0.009
Depression	Time	4.641	1	4.641	3.170	0.082
	Time* like or dislike	0.004	1	0.004	0.003	0.957
	Like or dislike	0.720	1	0.720	0.226	0.637
Anger–Hostility	Time	3.750	1	3.750	4.032	0.051
	Time* like or dislike	0.250	1	0.250	0.269	0.607
	Like or dislike	1.232	1	1.232	1.251	0.270
Vigor	Time	3.341	1	3.341	0.370	0.547
	Time* like or dislike	3.341	1	3.341	0.370	0.547
	Like or dislike	98.455	1	98.455	2.947	0.093
Fatigue	Time	4.926	1	4.926	1.236	0.273
	Time* like or dislike	0.017	1	0.017	0.004	0.948
	Like or dislike	0.614	1	0.614	0.055	0.816
Confusion	Time	21.684	1	21.684	17.217	<0.001
	Time* like or dislike	1.593	1	1.593	1.265	0.267
	Like or dislike	0.684	1	0.684	0.173	0.679
TMD	Time	751.705	1	751.705	15.914	<0.001
	Time* like or dislike	24.614	1	24.614	0.521	0.474
	Like or dislike	434.547	1	434.547	3.350	0.074

*interaction.

The results of the two-way repeated measures ANOVA showed a significant main effect of preference (like or dislike of singing) on “tension.” The result “tension” of the “dislike singing” group was higher than that of the “like singing” group before singing.

## Discussion

The results of this study demonstrate that singing can be effective in improving both the mental and oral condition of the elderly. In this study, 44 subjects (10 men and 34 women) who met the inclusion criteria were evaluated. The efficacy of singing on mental health status and immunocompetence was examined by the swallowing function, oral condition, blood, and saliva tests, as well as through questionnaires.

In the analyses of the swallowing function and mouth performance test results for all 44 subjects, a significant increase in saliva was noted in the unstimulated saliva test after singing compared with before singing. Moreover, in the saliva test, a significant decrease in the cortisol and sIgA levels was noted after singing compared with before singing.

Cortisol is a steroid hormone secreted from the zona fasciculata of the adrenal cortex and is most often studied in connection with stress. Moreover, cortisol affects the metabolic, immune, circulatory, and central nervous systems, and it is considered to be important for both mental and physical health [14]. The correlation of cortisol concentration in the saliva with that in the blood is approximately 0.90, so there is a strong correlation between the saliva cortisol level and cortisol in the blood [15]. The cortisol level increases in response to acute mental or physical stress. The cortisol concentration in saliva increases by from 50% to 100% due to acute changes in mental health status (i.e., giving a speech), with the peak of the increase lasting 20 to 30 minutes after the stress has dissipated [16]. A reduced sIgA level in the saliva has been reported to be related to the onset of upper respiratory tract infections [17]. The relationship between stress and sIgA has been examined in research studies using saliva samples since the early days of stress research. The sIgA concentration increases by 20% to 100% due to acute stress, such as mental arithmetic or a verbal presentation, and this increase lasts from just before the initiation of the stress to immediately after the end of the stress [18,19]. In contrast, although there are a limited number of reports on the subject, the sIgA concentration has been reported to fall depending on the type of stress, such as viewing a terrifying image or being immersed in cold water [19]. Additionally, the sIgA level was reported to increase after exercise of short duration [19].

The early phase of a change in sIgA concentration reflects the activity of the sympathetic nervous system and does not reflect a change in immune function, such as those carried out by B cells [20]. The relationship between sIgA and chronic stress has also been evaluated. For example, Bosch et al. [20] summarized seven studies on the relationship between sIgA concentration and stress and showed that a student’s sIgA concentration in saliva fell during examinations over a long period of time. Another study reported that the sIgA concentration remained low until 6 days to 2 weeks after the end of an examination period [21]. Taken together, the results from the present study show that the amount of saliva increased, while the salivary stress markers cortisol and IgA decreased, after singing. We believe these results indicate that singing has a relaxing effect.

From the VAS questionnaires, significant improvements were found in all of the following items after singing compared with before singing: “refreshed,” “comfortable,” “pleasurable,” “light-hearted,” “relieved,” and “relaxed.” The POMS questionnaires showed significant improvements in “tension,” “confusion,” and “TMD.” We found, therefore, that singing can be effective in improving the mental state of the elderly.

A differential analysis of the groups of subjects who responded that they “like singing” and “dislike singing” showed similar results for salivary amount, salivary test results, and questionnaire responses for all 44 subjects. Regardless of whether the subject answered “like singing” or “dislike singing,” the singing activity itself was considered to have contributed to improvement of the oral environment, as well as to the reduction of stress markers, except the RSST, in the saliva. Interestingly, this result indicates that we can expect greater benefits, with respect to the RSST, from singing by individuals who like singing. The results of the two-way repeated measures ANOVA showed a significant main effect of preference (like or dislike of singing) on the systolic blood pressure, pulse rate, “tension” of the POMS, and “refreshed,” “pleasurable,” and “relaxed” of the VAS. The pulse rate and “tension” of the POMS of the “dislike singing” group were higher than those of the “like singing” group before singing; and “refreshed,” “pleasurable,” and “relaxed” feelings of the “dislike singing” group were lower than those of the “like singing” group before singing. We think these results may be caused by differences in the mood of the subjects before singing. Because the subjects who like singing looked forward to singing, there is a possibility that the subjects who dislike singing were not looking forward to singing and thus felt tense prior to the task.

Based on the above results, singing seems to contribute to the improvement of the oral environment due to increased salivary secretion. Because stress markers in the saliva decreased and mood states of the VAS and POMS questionnaires improved, singing was shown to have contributed to stress mitigation. Therefore, singing may provide a positive impact on not only the oral environment, but also on stress relief. While significant effects were found with time, such as for the systolic blood pressure and pulse, we did observe a slight increase in blood pressure after singing. In future studies, we should confirm if this phenomenon is caused by the physical exertion of singing or by the elation resulting from singing. However, the average pulse rate of the subjects decreased slightly while singing. It is also important to assess if these results indicate a mental sedating effect. Significant effects of preference were found for the systolic blood pressure, pulse, and VAS, indicating refreshed, pleasurable, and relaxed feelings, but the POMS indicated tension. These results do not permit us to unequivocally assert that singing has a mentally stabilizing effect on a person who likes this activity. Because there were confounding factors, such as individual health conditions, we can only conclude that good health, an enjoyment of singing, and mental stability tend to be correlated.

Although no improvement was noted in swallowing function, immunity test (NK cell activity), and endocrine test (DHEA-s) results, these would be expected to be increased not by singing on just one occasion, but by continuous participation in singing. The results for lysozyme, chromogranin A, and amylase were inconclusive; however, significant differences were found in cortisol and S-IgA levels. We speculate that the reasons for these smaller than expected changes are the shortness of the observation time and limits to the sensitivity of these tests.

Because the results of this study show that one singing session allowed short-term, continuous improvement in mental health status and mood, further study is required to evaluate the effect of singing in daily life on body functions, including swallowing function and immune and endocrine conditions.

## Conclusions

In this study, the beneficial effects of singing on mental health status and immunocompetence were examined using swallowing function, oral condition, blood, and saliva tests, as well as through questionnaires taken before and after singing. The results of these tests suggest that singing can be effective in improving the mental and oral condition of the elderly.

## Competing interests

The authors declare that they have no competing interests.

## Authors’ contributions

KY conceived the study, participated in the design of the study, carried out data collection and drafted the manuscript. YT performed the statistical analysis. KR and NA and AH and AT and ES and KT participated in the design of the study and carried out data collection. IS looked over the study. All authors read and approved the final manuscript.
